# Eliciting and stop dose during oral food challenges for peanut and common tree nuts in different age groups

**DOI:** 10.1002/iid3.1152

**Published:** 2024-01-19

**Authors:** Wouter W. de Weger, Diede Jansen, Lidy van Lente, Gerbrich N. van der Meulen, Arvid W. A. Kamps

**Affiliations:** ^1^ Department of Paediatrics Martini Hospital Groningen The Netherlands; ^2^ Department of Epidemiology Martini Hospital Groningen The Netherlands

**Keywords:** allergy, processes

## Abstract

**Background:**

Oral food challenges (OFCs) are used to confirm or reject a diagnosis of food allergy. However, younger children may encounter difficulties in consuming all offered doses during an OFC in the absence of symptoms, resulting in inconclusive outcomes. Our aim is to assess the eliciting dose for objective symptoms among various age groups and determine the necessity of consuming the final dose step during an uneventful OFC to avoid false negative outcomes.

**Methods:**

OFCs for common food allergens performed between 2012 and 2019 were analyzed retrospectively. The primary outcome was the association of age with stop dose for OFCs with inconclusive outcome. Secondary outcome measures were the association of age with eliciting dose and the potential number of false negative outcomes.

**Results:**

A total of 1327 OFCs were performed in 707 patients. Of these, 514 (38.7%) were positive, 589 (44.4%) negative, and 224 (16.9%) inconclusive. In OFCs with inconclusive outcome, age appeared to be a significant predictor of the stop dose only for almond (*p* = .005). Objective symptoms occurred after the last dose step in 2%–13% of all OFCs with positive outcome. In our cohort, potential false negative outcomes may have been drawn in 27.6% of uneventful OFCs.

**Conclusions:**

Two third of children under 6 years of age successfully consumed all the provided doses during OFCs with a negative outcome. The eliciting dose for objective symptoms was not associated with age, and in a substantial number of OFCs with positive outcome, symptoms occurred after eating the final dose. These findings suggest that in case of an uneventful OFC, the outcome should be drawn only after a cumulative dose of 4.4 g has been consumed to avoid the risk of a potential false negative outcome.

## Key points

- Younger children may encounter difficulties in consuming all doses during an oral food challenge (OFC).
- Symptoms occur after the final dose in a substantial number of patients, irrespective of age.
- The final outcome of uneventful OFCs should be drawn after all doses have been consumed.

## Graphical abstract

We investigated the eliciting dose for objective symptoms among various age groups, and determined the necessity of consuming the final dose step during an uneventful OFC to avoid false negative outcome.

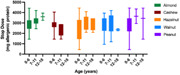

## 1 INTRODUCTION

Food allergy causes a significant, and most often lifelong burden for patients, emphasizing the importance of confirming or rejecting the diagnosis. This especially accounts for peanut and tree nuts, as these are hardly ever outgrown.^1^

Oral food challenges (OFCs) are essential to confirm whether one is allergic to a suspected food allergen. OFCs are performed to determine the nontolerated dose and severity of symptoms to give appropriate advice for individual patients how to avoid the specific allergen and prescribe medication (e.g., antihistamines and/or adrenaline auto‐injector).^2^, ^3^ To date, we rely on interpretation of symptoms to determine the outcome of an OFC. Unequivocal eliciting doses (EDs) can be determined if objective symptoms occur during an OFC. Establishing this reference dose may be more challenging in case only subjective symptoms occur (e.g., aversion, pruritis, nausea, and abdominal pain). According to guidelines, the challenge outcome is also positive in case subjective symptoms persist or occur repeatedly during successive dose steps.^4^, ^5^

In our daily practice, we frequently encounter situations where younger children did not consume the recommended total dose but the OFC was otherwise uneventful. These inconclusive outcomes present a dilemma: should we conclude that the OFC was terminated prematurely because the total amount was simply too much for a consumable portion for the child, or should we consider it indicative of aversion, suggestion a food allergy? In case of an inconclusive OFC, it is recommended to perform an additional challenge with a standard serving to confirm the outcome of the first performed OFC.^4^

To address the question whether the OFC outcome can be established if not all doses are consumed in younger children, it is essential to ascertain whether objective symptoms can manifest at each dose step. For example, the number of potential false‐negative OFC outcome (i.e., incorrectly diagnosed as tolerant) may increase if objective symptoms did not occur although the recommended total dose was not consumed. The guidelines recommend to administer a cumulative dose of 4.4 g of allergenic protein during the OFC based on the amount typically found in a standard serving.^5^, ^6^ A recent Dutch recommendation for OFC dosing schedules proposed using a lower total amount of 3.4 g of allergenic protein for children under the age of 12, aligning with age‐appropriate portion sizes (15 g of tree nuts or 17 g of peanuts).^7^ For peanut, an appropriate portion is considered to be 17 g of peanuts, which can be achieved in all available dosing schedules for specific age groups. While younger children may encounter difficulties in consuming all offered doses, to our knowledge, this aspect has not been formally studied.

Based on the assumptions outlined above, we conducted a retrospective analysis of a large cohort of children to evaluate the ED for objective symptoms, and also the ability to consume all offered doses during an uneventful OFC in different age groups. Our aim is to determine whether it is essential for younger children to consume the final dose step during an uneventful OFC to prevent a false negative outcome. We hypothesized that a cumulative dose lower than 4.4 g of allergenic protein cannot be used for children, regardless of their age, to confirm or reject the diagnosis of food allergy.

## 2 METHODS

### 2.1 Study population

This study was a retrospective cohort analysis in a pediatric allergy clinic of a large teaching hospital in the Netherlands. Children until 18 years of age who visited our outpatient allergy clinic for suspected food allergy between January 2012 and December 2018 were eligible for inclusion if an open OFC had been performed. Analysis was restricted to OFCs performed for peanut, hazelnut, cashew, walnut, and almond as these foods were challenged most frequently. If repeated OFCs were performed for a specific allergen in an individual patient, only the outcome of the first OFC was included. The Medical Ethical Committee of the Martini Hospital, Groningen, The Netherlands, has approved the study protocol (MEC 2020‐097). Informed consent was not required as this was a retrospective analysis of standard care. In clinical practice, parents are asked for consent once the recommendation to conduct an OFC has been made and the procedure has been thoroughly explained to them.

### 2.2 OFCs

Open OFC was conducted according to international guidelines.^4^, ^5^ The suspected food allergen was administered in seven or eight steps with a gradually increasing dose. These incremental series started with a dose of 1 or 3 mg, followed by 10, 30, 100, 300, 1000, and 3000 mg allergenic protein, resulting in a total of 4.443 or 4.444 g of allergenic protein. In case progressive and/or persistent subjective or mild objective symptoms occurred, patients were offered the same dose of allergenic protein according to protocol.^4^, ^5^ ED is defined as the amount of allergenic protein in milligrams at which objective symptoms appear for the first time. Stop dose (SD; allergenic protein in milligrams) is defined as the sum of all consumed doses at the moment OFC was ended. Symptoms were classified as objective or subjective according to predefined criteria (Appendix A: Table A1). The OFC outcome was classified as positive with objective symptoms if at least one objective symptom was registered. In case only subjective symptoms had occurred, the outcome was classified as positive with only subjective symptoms. OFCs during which no symptoms occurred were classified as uneventful. This category was then subdivided into OFCs with negative (in case all dose steps were consumed) and inconclusive outcome (not all dose steps were consumed), respectively. Patients were excluded in case the standardized incremental dose steps had not been followed. The following age groups were defined: 0–6, 7–11, and 12–18 years.

### 2.3 Outcome measures

The primary outcome measure was the association of age with SD for OFCs with inconclusive outcome. The secondary outcome measure was the association of age with ED (for OFCs with positive outcome) as well as the potential number of false negative outcomes.

### 2.4 Statistical analysis

Categorical variables were reported as numbers (percentages). Continuous variables were reported as mean (standard deviation) when normally distributed, or as median (interquartile range) if skewed. Correlation between age and SD as well as ED, respectively, was investigated using Pearson or Spearman test. Statistical analyses were performed using IBM SPSS Statistics version 25 (IBM). A *p* < .05 was considered statistically significant. GraphPad Prism Software was used to create all figures.

## 3 RESULTS

**Figure 1:**
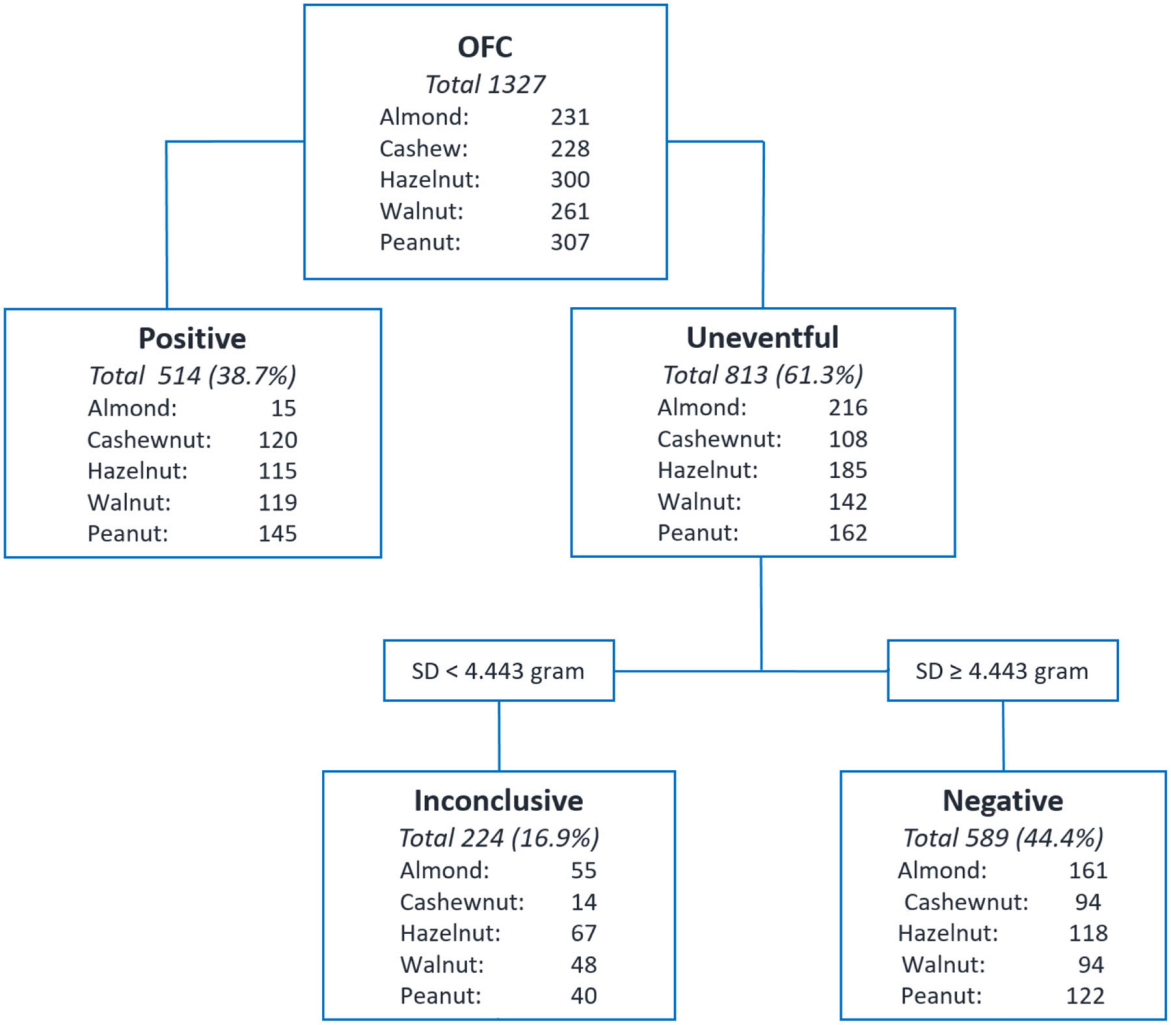
Flowchart of OFC outcomes. Percentages indicate percentage of total OFC. OFC, oral food challenge; SD, stop dose (the sum of all consumed doses [allergenic protein] at the moment OFC was ended) Uneventful; OFC during which no symptoms occurred. Negative; in case all dose steps were consumed and no symptoms occurred. Inconclusive; no symptoms occurred but not all dose steps were consumed.

A total of 1327 OFCs were performed in 707 patients. In 342 (48.4%) of these patients, an OFC for only one allergen was performed. In all other patients, OFCs for multiple allergens were performed. OFCs were performed 307 (23.1%) times for peanut, 300 (22.6%) for hazelnut, 261 (19.7%) for walnut, 228 (17.1%) for cashew, and 231 (17.4%) for almond. Characteristics of the patients are presented for each specific allergen in Table 1. Of all OFCs, 514 (38.7%) were positive, 589 (44.4%) negative, and 224 (16.9%) inconclusive (Figure 1). Antihistamines were administered 383 times (74.5%), and adrenalin 33 times (6.4%) in OFCs with positive outcome.

**Table 1:** Patient characteristics for OFCs performed for specific allergen.

	Peanut	Almond	Cashew	Hazelnut	Walnut
OFCs	307 (23.1%)	231 (17.4%)	228 (17.1%)	300 (22.6%)	261 (19.7%)
Female sex	111 (36.2%)	80 (34.6%)	95 (41.7%)	94 (31.3%)	107 (41.0%)
Age (y)	2.0 (1.0–4.0)	3.0 (1.0–7.0)	4.0 (2.0–8.0)	3.0 (1.0–7.0)	4.0 (2.0–9.0)
Eczema	227 (73.9%)	165 (71.4%)	142 (62.3%)	209 (69.7%)	162 (62.1%)
Asthma/recurrent wheeze	88 (28.7%)	91 (39.4%)	86 (37.7%)	116 (38.7%)	92 (35.2%)
Allergic rhinoconjunctivitis	25 (8.1%)	22 (9.5%)	27 (11.8%)	38 (12.7%)	44 (16.9%)
sIgE (kU/L)	*2.45* (*0.35–*>1*00)* a *15.8 (28.8)*	*0.79* (*0.35–*>*100)* b *4.7 (13.6)*	*1.5* (*0.35–>1005*)^c^ *7.1 (17.7)*	5.45 (0.35–>100)^d^ *20.3 (30.2)*	*1*.(*0.35–*>*100)3* e *6.0(15.4)*
Eliciting dose (mg)^f^	100 (1–3000)	30 (3–3000)	30 (1–3000)	100 (1–3000)	100 (1–3000)
	*281* (*524)*	*534* (*1099)*	*214* (*529)*	*433* (*745)*	*292* (*555)*
Stop dose (mg)^f^	444 (1–4444)	1443 (4–4444)	144 (3–4444)	444 (1–4444)	443 (2–4444)
	*1089* (*1412)*	*2301* (*1921)*	*886* (*1439)*	*1419* (*1683)*	*1173* (*1529)*
Positive family history of atopy	267 (87.0%)	204 (88.3%)	202 (88.6%)	261 (87.0%)	232 (88.9%)
One parent	144 (53.9%)	95 (46.6%)	104 (51.5%)	118 (45.2%)	113 (48.7%)
Both parents	68 (25.5%)	61 (29.9%)	55 (27.2%)	84 (32.2%)	56 (24.1%)
Sibling(s)	78 (29.2%)	66 (32.4%)	71 (35.1%)	82 (31.4%)	89 (38.4%)

*Note*: Values are presented as mean (SD), median (IQR) or number (%). Italics numbers represent mean (SD) of the eliciting and stop dose.

Abbreviations: IQR, interquartile range; OFC, oral food challenge.

^a^ 160/307 (52.1%).

^b^ 123/231 (53.2%).

^c^ 88/228 (38.6%).

^d^ 88/300 (29.3%).

^e^ 89/261 (34.1%).

^f^ these outcomes are for OFC with positive outcome.

### 3.1 SD

**Figure 2:**
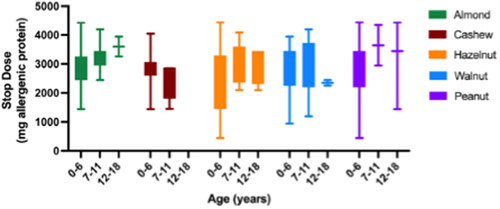
Stop dose of OFCs with inconclusive outcome. OFC, oral food challenge.

A total of 813 OFCs were uneventful (Figure 1). In 589/813 (72.4%) of all OFCs with an uneventful outcome, the children were able to consume all offered doses (cumulative dose ≥ 4443 mg of allergenic protein) and thus classified as “negative.” Among these OFCs, 375/589 (63.8%) were performed in children under 6 years of age (see Table 2). The OFC outcome was inconclusive (i.e., OFC uneventful but not all offered doses consumed) in 224/813 (27.6%) challenges (Figure 1). The SD for patients with an inconclusive outcome are presented per allergen for the different age groups in Figure 2. Specifically, 179/224 (79.9%) of these OFC were performed in children under 6 years of age (Table 3). In patients with an inconclusive OFC outcome, age appeared to be a significant predictor of the SD only for almond (*p* = .005; Appendix A: Table A2).

**Table 2:** Number of OFCs with negative outcome.

Age (years)	Almond	Cashew	Hazelnut	Walnut	Peanut
0–6 (63.8%)	115 (71.4%)	54 (57.4%)	71 (60.2%)	47 (50.0%	88 (72.1%)
7–11 (22.1%)	31 (19.3%)	29 (30.9%)	27 (22.9%)	23 (25.5%)	20 (16.4%)
12–18 (14.1%)	15 (9.3%)	11 (11.7%)	20 (16.9%)	23 (24.5%)	14 (11.5%)

*Note*: Data stratified for age groups.

Abbreviation: OFC, oral food challenge.

**Table 3:** Number of OFCs with inconclusive outcome.

Age (years)	Almond	Cashew	Hazelnut	Walnut	Peanut
0–6 (79.9%)	42 (76.4%)	10 (71.4%)	57 (85.1%)	35 (72.9%)	35 (87.5%)
7–11 (15.1%)	11 (20.0%)	4 (28.6%)	6 (9.0%)	11 (22.9%)	2 (5.0%)
12–18 (4.5%)	2 (3.6%)	0 (0.0%)	3 (4.5%)	2 (4.2%)	3 (7.5%)

*Note*: Data stratified for age groups.

Abbreviation: OFC, oral food challenge.

### 3.2 ED

**Figure 3:**
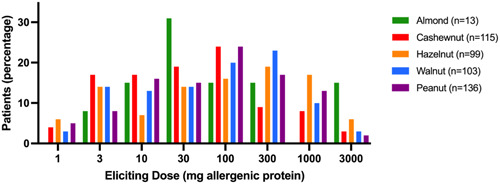
Eliciting dose of OFCs with positive outcome. OFC, oral food challenge.

**Figure 4:**
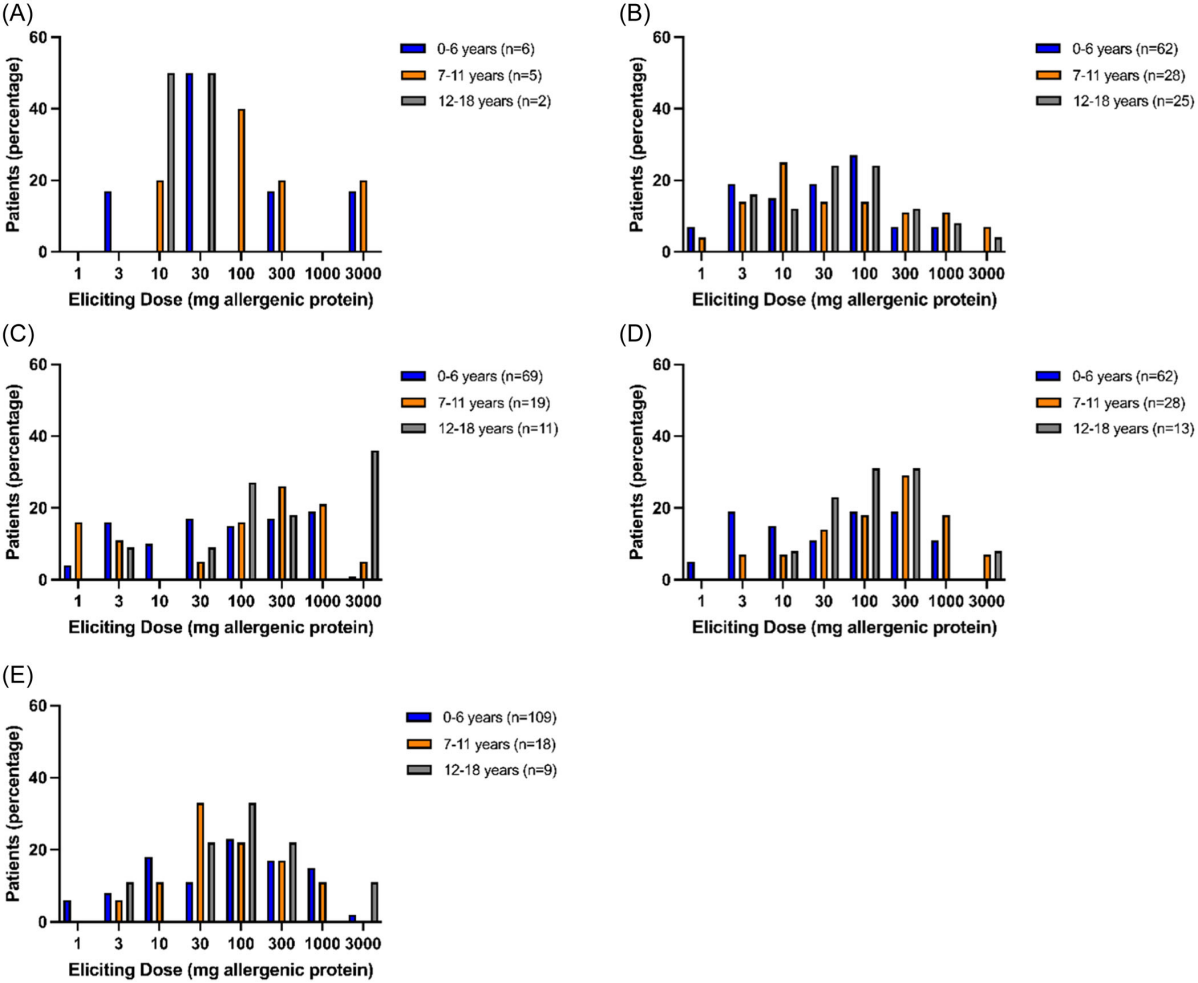
Eliciting dose of OFCs with positive outcome. Data presented for age groups. OFC, oral food challenge; a, almond; b, cashew; c, hazelnut; d, walnut; e, peanut.

The number of patients with a positive OFC outcome only after the final dose was consumed, varied from 12.4% to 40.0% depending on the allergen (Table 4). Objective symptoms occurred in the majority of positive OFCs (92.2%). ED for objective symptoms occurred after administration of the last dose of 3000 mg in 2%–13% of patients, depending on the allergen. Analysis of ED was performed for OFCs with positive outcome (Figure 3). The ED per allergen was not associated with age (Figure 4A–E and Appendix A: Table A3). Objective symptoms were elicited at each dose step for almond, hazelnut, and peanut. For cashew (Figure 4B) and walnut (Figure 4D) objective symptoms occurred only at dose steps below 3000 mg for children under 6 years of age.

**Table 4:** Number of patients with positive OFC only after final dose (cumulative dose ≥ 4.4 g) presented per allergen.

Food	Total dose ≥ 4.4 g
Almond	6/15 (40.0%)
Cashew	15/120 (12.5%)
Hazelnut	24/115 (20.9%)
Walnut	19/119 (16.0%)
Peanut	18/145 (12.4%)

Abbreviation: OFC, oral food challenge.

### 3.3 Potential false negative outcome

Based on the number of inconclusive challenges (i.e., uneventful OFC but not all dose steps consumed), we determined that a potential false negative outcome conclusion may have been drawn in 224/813 (27.6% of all patients with an uneventful OFC; see Figure 1). Specifically per allergen, the percentages for potential false negative OFC were 25.4% for almond, 12.9% for cashew, 36.2% for hazelnut, 33.8% for walnut, and 24.7% for peanut, respectively.

## 4 DISCUSSION

To our knowledge, this is the first study to determine the OFC outcome in various age groups. We demonstrated that almost two third of children under 6 years of age were able to consume all offered doses in case the OFC was uneventful (i.e., negative OFC outcome). This finding suggests that there is no reason to adjust OFC dosing schedules for younger children. Furthermore, in the majority of tests, objective symptoms occurred. For peanut, hazelnut, and almond, but not for cashew and walnut, objective symptoms were elicited during the last dose step. Our results imply that a firm conclusion about the OFC outcome may only be drawn if the last dose of 3000 mg was consumed (total ingested dose of 4.4‐g allergenic protein) to prevent a false negative outcome for peanut, hazelnut, and almond.

In our cohort, specifically for cashew and walnut, a definite outcome may have been established if the dose step of at least 1000 mg (total ingested dose of 3.3 g allergenic protein) was consumed. However, in a recent study by Dobbertin‐Welsch et al., it was shown that the whole spectrum of severity of symptoms occurred at every dose step for peanut, hazelnut, walnut, and cashew (almond was not included in their study).^8^ This difference may be explained by variation in the interpretation of symptoms and stop criteria between clinics, as has been stated before.^9^ Based on the results of our study, and in accordance with current guidelines, we recommend to establish the outcome to be negative, only if the test was uneventful after all dose steps have been consumed. If this is not achieved, an additional challenge for a single total dose of at least 4.4 g of the specific food allergen should be conducted during another visit.^4^ This way, potential false negative outcomes may be prevented.

It would have been interesting to evaluate whether patients with an inconclusive OFC were exposed to the specific allergen during follow‐up to determine if these patients were truly tolerant. However, we did not collect this information in a standard way. Furthermore, we should address the “lowest observed adverse effect levels” (LOAELs) and “no observed adverse effect levels” (NOAELs).^10^, ^11^, ^12^, ^13^ The LOAEL is the initial dose at which objective symptoms start to occur. The NOAEL is the preceding dose, thus the highest dose that does not lead to objective symptoms. LOAELs and NOALS are individual threshold doses for allergic reactions to occur, either discrete or cumulative. The true ED for objective symptoms lies in between the NOAEL and LOAEL, between the dose where objective symptoms are occurring and the prior dose. A similar nuance should be applied to the SD. In case mild or moderate symptoms occur, the last consumed dose may be repeated instead of proceeding with the subsequent dose step to prevent a more severe reaction. The true SD will, therefore, be more difficult to determine.

We evaluated eliciting and SD for open OFCs. This reflects our daily practice in which we mainly perform open OFCs for peanut and tree nuts, as these are more patient‐friendly, and cost‐efficient. Another advantage of an open OFC may be that a potential food anxiety is reduced simultaneously since patients know what they are eating. This may facilitate easier introduction of the food at home in case the OFC outcome is negative.^4^, ^7^ By performing double‐blind placebo controlled food challenges (DBPCFCs) we might have been able to detect a placebo reaction more easily which would potentially result in different ED and SD. On the other hand, in the majority of OFCs in our study, objective symptoms occurred.

Several studies have been undertaken to assess the efficacy of a single or low‐dose OFC. This approach proves valuable in establishing a population‐level threshold dose for a specific allergen.^14^, ^15^ Additionally, for individual patients with a food allergy, confirmation of tolerance for a low‐dose OFC can potentially mitigate the impact of a stringent elimination diet.^16^ However, as we have demonstrated, a substantial portion of the patients react after ingesting a higher dose of a specific allergen. Conducting a low‐dose OFC for these particular patients poses a potential risk, as there is a chance they might react in daily life when consuming a regular/higher portion.

The following limitations should be addressed. First, since our analysis was restricted to open food challenges, this recommendation may not apply for double‐blind placebo‐controlled food challenges (DBPCFCs). Nevertheless, it is reasonable to assume that younger children may encounter challenges in consuming the total amount administered during DBPCFCs due to larger portion sizes, as the suspected food allergen is concealed within other foods (e.g., gingerbread). Therefore, additional research is warranted to investigate whether our findings also apply to DBPCFCs.

Second, it is questionable whether our results can be extrapolated to other centers as it has been reported that there is variation in the interpretation of symptoms and stop criteria. This may be due to differences in the interpretation of guidelines or fear of a severe reaction to occur.^9^ Third, we did not analyze the severity of symptoms. However, Dobbertin‐Welsch et al. recently reported that the whole spectrum of severity of symptoms occurred at every dose step except for anaphylaxis.^8^ In their study, one out of three patients presented with symptoms of the lower respiratory tract indicating signs of anaphylaxis, and one‐third of all patients developed symptoms at higher dose levels with more severe allergic reactions according to severity grade IV. Finally, previous allergic reactions and specific IgE level may be associated with the OFC outcome. However, recent specific IgE values and/or information about allergic events were not always available due to the retrospective design of this study.

In conclusion, the results of this study demonstrate that the majority of children under the age of 6 years were able to consume all offered doses during uneventful OFCs (i.e., negative OFC outcome) for peanut and common tree nuts. Also, the ED for objective symptoms was not associated with age, and these symptoms occurred after the last dose step in a substantial number of OFCs with positive outcome. These findings underscore that in case of an uneventful OFC, the outcome should only be drawn after a final dose of 3000 mg has been consumed (i.e., cumulative dose of 4.4 g) to prevent a potential false negative outcome.

## AUTHOR CONTRIBUTIONS

**Wouter W. de Weger**: Conceptualization; data curation; formal analysis; writing—original draft. **Diede Jansen**: Data curation; formal analysis; writing—original draft. **Lidy van Lente**: Conceptualization; formal analysis; methodology; writing—review and editing. **Gerbrich N. van der Meulen**: Writing—review and editing. **Arvid W. A. Kamps**: Conceptualization; methodology; supervision; writing—review and editing.

## Data Availability

The data that support the findings of this study are available from the corresponding author upon reasonable request.

## APPENDIX A

### A.1

**Table A1:** Objective and subjective symptoms.

	Objective	Subjective
Skin/mucosa	Erythema	Pruritis/scratching
	Exanthema	Itchy mouth
	Eczema	
	Urticaria	
	Edema	
	Swelling (tongue, lips, face, eyes)	
	Conjunctivitis	
Gastrointestinal	Vomiting	Dysphagia
	Diarrhea	Nausea
		Abdominal pain/cramps
		Drooling
Respiratory	Stridor	Perception of throat tightness
	Dyspnea	
	Coughing	
	Rhinitis	
	Sneezing	
Cardiovascular/neurological		Dizziness
General		Pallor
		Change of behavior (e.g., agitation)
		Crying

**Table A2:** Association of stop dose with age for OFCs with inconclusive outcome.

Food	*p* Value
Almond (*n* = 55)	**.005***
Cashew (*n* = 14)	.424
Hazelnut (*n* = 67)	.072
Walnut (*n* = 48)	.347
Peanut (*n* = 40)	.118

*Note*: Asterix indicates significant value.

Abbreviation: OFC, oral food challenge.

**Table A3:** Association of eliciting dose with age for OFCs with positive outcome.

Food	*p* Value
Almond (*n* = 15)	.806
Cashew (*n* = 120)	.070
Hazelnut (*n* = 115)	.230
Walnut (*n* = 119)	082
Peanut (*n* = 145)	.594

Abbreviation: OFC, oral food challenge.
